# ‘I’m the only mum she knows’: parents’ understanding of, and feelings about, identity-release egg donation

**DOI:** 10.1093/humrep/deac174

**Published:** 2022-08-25

**Authors:** J Lysons, S Imrie, V Jadva, S Golombok

**Affiliations:** Centre for Family Research, University of Cambridge, Cambridge, UK; Centre for Family Research, University of Cambridge, Cambridge, UK; Thomas Coram Research Unit, University College London, London, UK; Centre for Family Research, University of Cambridge, Cambridge, UK; Institute for Women’s Health, University College London, London, UK; Centre for Family Research, University of Cambridge, Cambridge, UK

**Keywords:** egg donation, identity-release, ART, donor linking, qualitative research

## Abstract

**STUDY QUESTION:**

How do parents understand and feel about identity-release egg donation?

**SUMMARY ANSWER:**

Almost one-third of mothers and fathers did not understand the identifiable nature of their egg donation; mothers expressed complex and sometimes difficult feelings about the prospect of future donor–child contact.

**WHAT IS KNOWN ALREADY:**

Identity-release egg donation has been the only treatment option available to patients wishing to pursue this route to parenthood in the UK since 2005. However, little is known about how well parents understand this legislation, and how they feel about potential donor–child contact.

**STUDY DESIGN, SIZE, DURATION:**

This qualitative interview study included 61 mothers and 51 fathers whose 5-year-old children were conceived via identity-release egg donation. Interviews were conducted between April 2018 and December 2019.

**PARTICIPANTS/MATERIALS, SETTING, METHODS:**

Data are reported from phase two of a longitudinal study of families created using open-identity egg donation. In-depth, semi-structured interviews were conducted with mothers and fathers. The interviews contained a section on what parents understood about the identifiable nature of the donor. These data were analysed using qualitative content analysis. Mothers who understood the identifiable nature of their egg donation (n =* *44) were then asked about their thoughts and feelings regarding the prospect of future donor–child contact. Mothers’ narratives were analysed using thematic analysis.

**MAIN RESULTS AND THE ROLE OF CHANCE:**

Almost one-third of parents (28% of mothers, n = 17; 31% of fathers, n =* *16) did not understand the identifiable nature of their egg donation. Mothers’ and fathers’ misunderstandings about identity-release egg donation fell into two categories: (i) Unclear about identity-release and (ii) Belief that the donor is anonymous. Reflexive thematic analysis revealed that egg donation mothers’ feelings about identity-release donation could be understood via three organizing themes: (i) *identity-release as a threat*, (ii) *acceptance: it is what it is* and (iii) *embracing identity-release.* The findings indicated that egg donation mothers utilized various strategies to manage their feelings about identity-release egg donation in day-to-day life, and each theme was associated with at least one coping strategy.

**LIMITATIONS, REASONS FOR CAUTION:**

Participants were predominantly from White, middle-class backgrounds. Further research with a more diverse sample is needed to improve generalizability.

**WIDER IMPLICATIONS OF THE FINDINGS:**

These findings indicate that parents would benefit from more comprehensive provision of information, both at time of treatment and following conception, to ensure they have fully understood the nature of the donation. Parents may also benefit from follow-up care to help manage any complex or difficult feelings about donor–child contact.

**STUDY FUNDING/COMPETING INTEREST(s):**

This research was supported by a Wellcome Trust Collaborative Award [208013/Z/17/Z]. The authors have no conflicts of interest to declare.

**TRIAL REGISTRATION NUMBER:**

N/A.

## Introduction

Egg donation is an increasingly common form of fertility treatment offered to women who are unable to conceive using their own eggs. Recent years have seen an increase in the number of families created using egg donation, with the number of IVF cycles using donor eggs in the UK rising from 2263 in 2014 to 4212 in 2018, representing 6.1% of all IVF cycles in 2018 (Human Fertilisation and Embryology Authority, 2020). Legislation removing donor anonymity was introduced in the United Kingdom in 2005; those wishing to become parents using egg donation must therefore use an identifiable donor. This reflects a global trend towards greater openness in ART, with many (e.g. Sweden, Germany, Norway, New Zealand and Australia) although not all (e.g. Spain, Israel and China) countries enforcing legislation that prohibits donor anonymity in recent decades. Thus, in the UK, patients can choose a donor who is known to them, or, more commonly, an identity-release donor supplied by the clinic. With identity-release donation in the UK, the donor is anonymous to intended parents at the time of treatment, but the child has the right to access identifying information about the donor from the age of 18. Identity-release legislation was introduced following a wide-ranging public consultation and enables those who wish to, and who have been informed about the nature of their conception, to find out the identity of their donor.

Concerns have been raised about the impact on family functioning of the prospect of future donor–child contact, as the donor may be perceived as an ongoing, salient presence within family life (Scheib *et al.*, 2000; Lampic *et al.*, 2014). Despite the increasing use of identity-release donation, very little is known about parents’ understanding of, and feelings about, this type of donation. The present study aimed to ascertain how egg donation parents think and feel about identity-release donation. As the first UK cohort of identity-release offspring will turn 18 in 2023, gaining an understanding of parents’ comprehension of the implications of identity-release legislation is particularly timely.

Data on parents’ feelings about donation type comes largely from studies of patients who had used anonymous or known donation. Some prospective parents chose anonymous donation to establish explicit boundaries between the donor and their family, and to avoid any potential legal issues (Hershberger *et al.*, 2007; Laruelle *et al.*, 2011). Parents who opted for anonymous donation have reported being motivated by a desire to minimize the role of the donor in their child’s conception story (Baetens *et al.*, 2000; Greenfeld and Klock, 2004), to limit the donor’s perceived intrusion into family life (Hudson, 2020), and to protect the mother–child relationship (Laruelle *et al.*, 2011). Some women opted for anonymous donation specifically to avoid the prospect of future donor–child contact (Greenfeld and Klock, 2004; Hershberger *et al.*, 2007). Access to information about the donor has been represented by some as a burden, threatening the ‘emotional distance’ from the donor that some mothers wished for (Rubin *et al.*, 2015). This is echoed by findings from a small UK-based study, which found that some mothers viewed the prospect of a known donor as contributing to a picture of ‘long-term insecurity’ (Stuart-Smith *et al.*, 2012).

Conversely, some parents have reported feelings of trepidation when considering the unknown origins of an anonymous donor, instead finding access to detailed donor information reassuring (Baetens *et al.*, 2000; Winter and Daniluk, 2004; Laruelle *et al.*, 2011). Such information enabled some mothers to feel that they had a bond or relationship with the donor (Hudson, 2020). Rubin *et al.*’s (2015) study of prospective egg donation parents found that, based on the information provided to them, mothers would look out for signs of a connection with the donor to build a satisfying narrative about her. Similarly, those opting for intra-family donation have cited the genetic and social connections between the mother and donor as a comforting feature, enhancing their feeling of connectedness to the child and increasing their feelings of kinship within the wider family network (Greenfeld and Klock, 2004; Jadva *et al.*, 2011; Imrie *et al.*, 2020).

Little information exists about how these considerations regarding known and anonymous donation may apply to those using identity-release donor eggs. Identity-release donation may pose specific challenges to prospective parents, as it could potentially combine the drawbacks of both anonymous and known donation. It may, therefore, represent an uncertain middle ground where parents must manage both the psychological and practical challenges of having very little information about the donor at time of treatment, alongside the knowledge of potential future donor–child contact (Imrie *et al.*, 2019a). A qualitative study of 11 women who had either received, or were waiting to receive, treatment in the UK with identity-release donor eggs found that some women felt that, as identity-release donors are unknown to the children throughout childhood, identity-release donation posed less threat to the security of the mother–child relationship than known donation (Stuart-Smith *et al.*, 2012). However, whilst not knowing about the donor was seen as protective by some mothers, the lack of information about the donor raised concerns for others. In the absence of information about the donor, there was a tendency for mothers’ perceptions of the donor to polarize, with some idealizing, and others demonstrating extreme wariness of, the donor.

At the first phase of the current study, the perspectives on non-genetic motherhood of 85 egg donation mothers were examined when the children were aged between 6 and 18 months (Imrie *et al.*, 2020). The study found that some mothers selected identity-release over known donation as a way of establishing more explicit boundaries between the donor and the family, and to minimize feelings of threat to the mother–child relationship. Having less information about the donor at the time of treatment helped mothers to ‘fully own’ the identity of being the child’s parent. However, no information was obtained on mothers’ feelings about future contact between the donor and the child.

It has been suggested that the potential for the child to obtain the identity of the egg donor may put pressure on the mother–child relationship, due to the absence of a genetic link between the mother and the child (Lampic *et al.*, 2014). To date, no research has specifically addressed this question with families formed through identity-release egg donation. A qualitative study of 23 parents whose adult offspring had obtained information about their sperm donor described the different strategies parents used to manage the presence of the donor in their lives (Widbom *et al.*, 2021). These included positioning the donor at a distance, or acknowledging the donor as a person, or even as part of the family. Fathers, in particular, were found to maintain a distance between themselves and the donor, often avoiding conversations about, and communication with, the donor. Some fathers demonstrated comfort in discussing topics around sperm donation more generally, but discomfort in discussing the donor as a person; the authors suggested that the absence of a genetic link between the father and child was perceived as a threat by some fathers when confronted with the reality of their child meeting their donor. This shows that, for sperm donation parents at least, the identity-release process can be experienced in a diversity of ways, from challenging the fathers’ role as a parent, to representing a source of identity information about their child. However, the extent to which these findings may be applicable to egg donation parents remains to be seen.

Given that identity-release donation has now been practiced in the UK for over 15 years, there remains a dearth of research examining how parents understand and experience this type of donation. Parents’ feelings about identity-release egg donation are particularly pertinent during early childhood as, in the UK, clinics and regulatory bodies have assumed a pro-disclosure stance, encouraging parents to begin telling their children about their method of conception around the age of 4 or 5 years (Human Fertilisation and Embryology Authority, 2021). It is possible that the prospect of starting the disclosure process may bring the implications of identity-release legislation into sharper focus for some parents. This may be particularly true for egg donation mothers, due to the double burden of the lack of a genetic link with the child, and the fact that mothers have been found to take most responsibility for disclosure in donor gamete families (Paul and Berger, 2007; Blake *et al.*, 2010). The present study aimed to understand parents’ knowledge, thoughts and feelings about identity-release donation when their child was aged 5 years—an age when many parents are beginning to tell their child about their donor conception.

## Materials and methods

### Sample characteristics

The present study examined families who had children conceived via identifiable egg donation. The families were originally recruited as part of a larger, longitudinal investigation of heterosexual couples who had conceived via IVF using either their own gametes, or donor eggs (Imrie *et al.*, 2019a, 2019b, 2020). The original sample of 85 egg donation families, and a comparison group of 65 own-gamete IVF families, were recruited through 12 fertility clinics in the UK; details of recruitment have been reported elsewhere (Imrie *et al.*, 2019b). At time one, all families provided written consent to be contacted again at phase two. Families were contacted when their child approached the target age of 5 years, between March 2018 and November 2019, and were asked to participate in the follow-up study.

Sample characteristics are presented in Table I. Seventy-two egg donation families participated at time two, representing an overall retention rate of 85%. Of the 72 egg donation families included at this phase, 63 (88%) had used identity-release donation and 9 (13%) had used known donation. The present paper reports on families who had used identity-release egg donation only. Two mothers were unable to participate in this section of the interview, and not all fathers were available for interview, making a total sample of 61 mothers and 51 fathers. Most mothers (71%) and fathers (71%) had undertaken higher education; all mothers and most (97%) fathers identified their ethnicity as White British. All mothers and fathers were either married or in non-marital cohabiting relationships at phase one; the majority (93%) of couples were still in intact relationships at phase two.

**Table I:** Family sociodemographic information.

	Identity-release egg donation (n = 61)	
	Mean	SD
Child’s age (months)	67.5	4.08
Mother’s age (years)	47.3	4.37
Father’s age (years)	48.6	6.42

	N (%)	

*Sex of child*		
Female	31 (51)	
Male	30 (49)	
*Mother’s education*		
School education	18 (30)	
Higher education	43 (70)	
*Fathers’ education*		
School education	15 (29)	
Higher education	36 (71)	
Couple relationship status		
Married	49 (80)	
Non-marital cohabitation	8 (13)	
Separated/divorced	4 (7)	

### Interviews

Each parent was interviewed at home by one of a team of trained researchers. In-depth, semi-structured interviews were conducted with mothers and fathers separately. Parents’ level of understanding about identity-release donation was ascertained by their answers to the questions ‘Is there anything else you or your child will be able to find out about the donor in the future?’ and ‘Is there anything you plan on telling your child specifically about the donor in the future?’ Parents who understood the identifiable nature of their egg donation were then asked about their thoughts and feelings regarding the prospect of future donor–child contact. Written informed consent was obtained from all participants and ethical approval was obtained from the University of Cambridge Psychology Research Ethics Committee.

### Analysis

The analysis was carried out in two phases. In Phase 1, qualitative content analysis was conducted to examine parents’ level of understanding about identity-release donation, as this approach is particularly appropriate for organizing responses to a particular question or relating to a specific theme (Mayring, 2015; Graneheim *et al.*, 2017). This technique enables the exploration of participants’ thoughts and experiences via the creation of categories that describe the participants’ responses, whilst remaining close to the data (Sandelowski, 2000; Schreier, 2012). Transcripts were first read, and initial codes were generated. These codes were then refined into two overarching categories, each of which contained sub-categories that represented a different type of understanding of identity-release egg donation. The transcripts were then coded according to these categories, and counts were made of each category code for both mothers and fathers. This approach is in line with previous research on experiences of family life following ART (Blake *et al.*, 2010; Zadeh *et al.*, 2017).

Phase 2 focused on mothers’ thoughts and feelings about identity-release donation. Given that mothers are the parents who lack a genetic link with the child, and they are also the parents who are most likely to discuss donor conception with the child, only the mother’s interview transcripts were analysed for this phase of the analysis. Forty-four mothers’ interview transcripts were coded, representing all of those who had a full understanding of identity-release. Pseudonyms were used to protect participants’ identities, and all identifying information was removed from the transcripts. The transcripts were analysed according to the principles of reflexive thematic analysis (RTA, Braun and Clarke, 2019), a method used for the identification and analysis of patterns within a dataset, enabling the researcher to construct themes that are salient in relation to the research question (Braun and Clarke, 2021). RTA is a multi-stage process, during which features of a dataset are systematically coded, sorted into themes, reviewed, refined and named (Braun and Clarke, 2021). The refining process should result in themes that are both discrete from each other, and also broad enough to represent various codes from different parts of the dataset (Attride-Stirling, 2001). The analysis was inductive and data-driven, so that resultant themes were closely linked to the data themselves. Theme generation was the result of a flexible, rigorous and recursive process, during which data audits were conducted to periodically assess the quality of the analysis process and to ensure that the thematic map was fully representative of the dataset. The final thematic map demonstrated 3 organizing themes and 11 subthemes.

As the results of RTA are considered generally accessible to the lay public, it is particularly suited to informing policy and procedure (Braun and Clarke, 2006). The present study is the first to examine parents’ perspectives on identity-release donation since the introduction of this legislation in the UK; as such, this analytical approach was deemed particularly appropriate.

## Results

### Parents’ understanding of identity-release egg donation

Forty-four (72%) mothers demonstrated at least a basic understanding of identity-release egg donation; however, 17 (28%) mothers did not understand that their egg donor would be identifiable to their child in the future. Thirty-five fathers (69%) understood the principles of identity-release donation, whilst 16 (31%) did not. Categories and illustrative quotations are presented in Table II. Mothers’ and fathers’ misunderstandings about identity-release egg donation fell into two categories: (i) Unclear about identity-release and (ii) Belief that the donor is anonymous, each with two sub-categories.

**Table II:** Proportions of parents who did not understand identity-release donation, and types of misunderstanding about identity-release donation.

	Mothers (n = 61)	Fathers (n = 51)	
Understands ID-release	44 (72%)	35 (69%)	
Does not understand ID-release	17 (28%)	16 (31%)	

Type of misunderstanding about ID-release	Mothers (n = 17)	Fathers (n = 16)	Examples of misunderstanding

**1. Unclear about ID-release**			
(1a) No awareness of ID-release	7 (41%)	11 (68%)	‘Well, I don’t really know much about the donor to be able to tell him anything, but I don’t-, I mean I think I would have to do more research before I told him anything, because I don’t know whether he’s entitled to find out about the donor, I don’t know how that works …’ *Mother* ‘Erm … I don’t know actually, I seem terribly remiss about this and I can’t remember, and I can’t even remember sort of whether these things are literally kind of closed off or whether it’s one of those that you can go back to the clinic and they have to keep records and, you know, you can … insist on finding out.’ *Father*
(1b) Partial/incomplete understanding of ID-release	3 (18%)	2 (13%)	‘When she’s 18 and she’s … I can’t remember what they said now about whether … do we tell her that she … I’ve forgotten all of that information … erm, because you have to by law here in this country, they have to know that … or they have to have access to information about having used donor eggs […] I think we have to though, I think legally we have to tell her’. *Mother* ‘I’m not sure what legal right she will have when she turns say eighteen to get further information. I don’t know’. *Father*

Type of misunderstanding about ID-release	Mothers (n = 17)	Fathers (n = 16)	Examples of misunderstanding

**2. Belief that donor is anonymous**			
(2a) Donor is anonymous	6 (35%)	2 (13%)	‘It’s all … it’s confidential, so you can never know who she was anyway …’ *Mother* ‘I don’t think it’s a possibility [to find out donor’s identity], but it would be very interesting’. *Father*
(2b) ID-release was/will be introduced after child was born	1 (6%)	1 (6%)	‘I don’t know because the law changed- when did it, it was kind of anonymous that, but now I think you can get information—I might be wrong about all this—but I think that when they’re 18 they’re allowed to find out about the donor if you want to tell them. But I can’t remember what, if that’s just recent, a recent thing, I think it was after we had him …’ *Mother* ‘I was reading something that they’re looking to bring in some legislation where you can actually find out, or rather if you wanted to, I guess if the child wanted to, you can find out the identity of the egg donor, but I don’t know where that is, I just saw a story about it’. *Father*

### Unclear about identity-release

#### No awareness of identity-release

Of the 33 parents who did not understand identity-release donation, most mothers (41%, n =* *7) and fathers (68%, n =* *11) did not know whether their child could access further information about the donor in the future. Some parents phrased this in terms of not being able to remember or needing to do some ‘research’ by looking at their medical paperwork from the treatment stage. Others said they did not know because they had not investigated the question, with one mother stating that she had not found out what information about the donor her child was entitled to as she did not intend to tell her child about her method of conception.

#### Partial understanding of identity-release

A small number of parents (18%, n =* *3 of mothers, 13%, n =* *2 of fathers) demonstrated vague but incomplete knowledge about identity-release donation. For example, two parents knew that age 18 is a threshold for children to seek out information, but they were unsure of precisely what information they would be entitled to access.

### The donor is anonymous

#### Belief that the donor is anonymous

This was the second most common type of misunderstanding about identity-release egg donation. Thirty-five per cent (n =* *6) of mothers and 13% (n =* *2) of fathers believed that their egg donor was fully anonymous and that their child would be unable to find out any further information about the donor.

#### Identity-release donation was introduced after child was born

One mother and one father demonstrated clear knowledge of identity-release donation but thought that the change in UK law from anonymous to identity-release donation had been introduced after their child was born. These parents, therefore, believed that identity-release legislation did not apply to their families and that their child would not be entitled to access identifying information about their donor in the future.

### Mothers’ thoughts and feelings about identity-release

**Figure 1.**
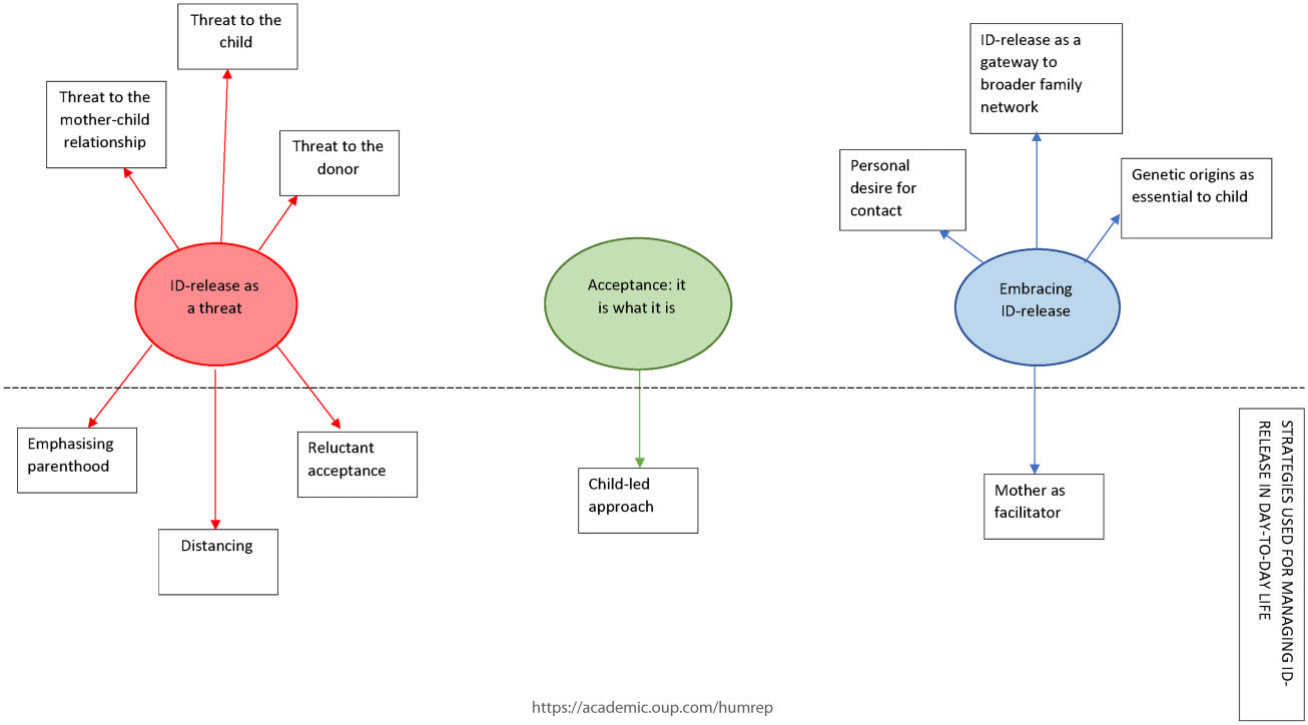
**Thematic map demonstrating relationships between themes and subthemes**.

This analysis included the mothers who understood what was meant by identity-release donation only. Three organizing themes were produced from the analysis: (i) *identity-release as a threat*, (ii) *acceptance: it is what it is* and (iii) *embracing identity-release.* The findings indicated that egg donation mothers utilized various strategies to manage their feelings about identity-release egg donation in day-to-day life, and each theme was associated with at least one coping strategy. Organizing themes and subthemes are depicted in a thematic map in Fig. 1. The organizing themes were not mutually exclusive; it was not uncommon for mothers to express multiple perspectives in their narratives, simultaneously perceiving the donor’s identity as a threat whilst also acknowledging the ability to trace the donor as an essential opportunity for their child.

### Identity-release as a threat

Many mothers viewed the prospect of knowing the donor’s identity, and potential donor–child contact, as threatening to some degree. This perceived threat was represented in three ways: as a *threat to the mother–child relationship, a threat to the child* and a *threat to the donor.* Mothers adopted at least one of three strategies to cope with this threat: *emphasizing parenthood, distancing* and *reluctant engagement.* Mothers adopted one or all of these strategies, although those who principally adopted a ‘reluctant engagement’ strategy were less likely to adopt a ‘distancing’ strategy, and vice versa.

#### Threat to the mother–child relationship

A prominent perspective was that the child’s ability to access the donor’s identifying information constituted a direct threat to the mother–child relationship. Identity-release was represented as having the power to weaken, or interfere with, mothers’ sense of ownership of their child, and as posing a challenge to their identity as a mother. One mother explained that her child’s interest in the donor would be an indicator that she’d ‘*not done my job properly*’, whilst another expressed a deep fear that the donor–child relationship would be more ‘real’ than the one she shared with her child:

‘I’d be worried that they’d suddenly have this unbelievable connection that perhaps is truer of a biological mother, and perhaps what we’ve got isn’t a true mother-son relationship? I don’t know, it worries me’. (Coleen)

Many mothers expressed the hope that their child would not seek contact with the donor in the future and stated that any interest their child was to show in the donor would be emotionally painful to them. Some mothers also expressed a fear of rejection, not just at the personal but also at the family-wide, level: ‘*probably a fear of mine really, is that he might really like her and her family and, like, in future Christmases decide to spend time with her rather than us’. (Wendy)*

Conversely, other mothers were concerned about the donor’s interest in the child. For example, one mother said that despite her curiosity about the donor, she would not want her child to initiate the identity-release process in case it piqued the donor’s interest in her child. Mothers were concerned that the identity-release process itself would have a negative, potentially long-term, impact on their relationship with their child. For example, Valerie felt that the prospect of identity-release influenced her parenting due to the fear that her child would one day use it as a weapon against her:

‘That’s probably why I nurture and care for her so much more, because I want her to be, to know that I, you know, absolutely adore the ground she walks on. I don’t want her to then say ‘well you were rubbish, I’m going to go and find who’s biologically mine’ or whatever. I want to overcompensate I suppose because I don’t want her to go and find [the donor]’.

Some mothers also worried that access to information about the donor’s identity or physical appearance may ‘*limit or influence*’ their own perception of their child, with concerns that this may then impact their relationship with their child.

#### Threat to the child

Identity-release was perceived by some as posing a threat to their child. Mothers expressed concerns about how the donor may behave, or what the donor might want from their child after making contact. Mothers worried that the donor might ‘*… take advantage of him in some way or hurt him*’. A related concern for several mothers was that they would have a personal reaction to the donor and that these reactions might disrupt their child’s experience of contact with the donor. This ranged from one mother creating an ‘*ideal vision*’ of the donor to mothers worrying that they may dislike the donor upon making contact. Others focussed on the possibility that their child may be disappointed or face rejection from their donor after attempting to make contact:

‘I wouldn’t like to rule [donor contact] out completely, but then obviously you couldn’t guarantee that’s what she was going to get, and she could be rebuffed and get really upset’. (Agatha)

Mothers also anticipated the identity-release process taking a toll on their children as young adults. Many thought that the process may be difficult or ‘*upsetting*’ for their child, and they expressed regret that their child may have to go through a difficult process. For some, there was a tension between advised early disclosure of identity-release donor conception to their child, and their child’s inability to access the donor’s identifying information until the age of 18. These mothers implied that this waiting period may cause their child emotional or psychological distress. These concerns were often compounded by the fact that because the identity-release system is so new in the UK, very little is known about the rates and quality of donor–child contact. Mothers, therefore, expressed a fear of the unknown, observing that ‘*even the experts don’t know how it would go*’.

#### Threat to the donor

Though less prominent, this subtheme relates to some mothers’ concerns that the identity-release process might negatively impact the donor’s life or discourage potential donors altogether. For several mothers, the potential for donor offspring to ‘*suddenly appear*’ in the donor’s life ‘*out of nowhere*’ was seen as a threat hanging over the donor’s future. Similarly, some mothers viewed not initiating the identity-release process as a way of expressing gratitude to their donor; the value of the donor’s gift was so great that despite their curiosity, mothers wanted to ‘*do the right thing … for her*’ and respect the donor’s boundaries and not ‘*rock the boat*’.

#### Strategy subtheme: emphasizing parenthood

Three strategy subthemes were identified as ways in which mothers managed their feelings of threat in day-to-day life. The first strategy was to emphasize the role of parenthood, thereby minimizing the impact that the donor’s identity could have on the parent–child relationship. This was expressed in several different ways; most commonly, the biological relationship shared by mother and child (through gestation) was considered to counteract, or neutralize, the threat of any potential bond between the child and the donor. Many mothers directly contrasted identity-release egg donation with adopted children seeking out their birth families, believing that because the donor did not gestate or give birth to them, their child would feel no imperative to seek a connection with them in the future. Mothers also referred to the experience of parenting as a protective factor; some expressed fears of their child developing a strong bond with the donor, but then reassured themselves by referring to their experiences of carrying and parenting their child. Noreen observed that:

‘I know that there will come a time where [child] legally will gain the right to gain access to the information. Part of me worries about that. And I think that I try to say to myself that this is stupid because I’m the only mum she knows. I’ve done everything for her, I carried her, I’ve given birth to her, I’ve done everything for her. I’m the only mum she knows’.

The belief that the experience of parenting trumps the genetic connection shared by donor and child was further underlined by the view taken by a small number of mothers who suggested that the donor’s experiences of parenting her own children would negate any desire to build a relationship with her donor offspring. Some mothers also believed that the quality of the mother–child relationship was a key factor in determining whether their child would wish to pursue the donor’s identity in the future. Whilst this strategy was comforting for most mothers, a few remained concerned that changes in mother–child relationship quality as children entered adolescence would result in their child’s increased interest in seeking the donor’s identity.

#### Strategy subtheme: distancing

A second strategy employed by mothers to manage their feelings of threat was to distance themselves from identity-release. Some mothers distanced themselves from the donor as a person, suggesting that ignorance of the donor’s identity was protective in some way. For example, one mother described her curiosity about the donor as ‘*dangerous*’, viewing identifying information about the donor as a potential threat to her psychological well-being. Other mothers expressed the view that it was ‘*important that you don’t know too much*’, to avoid knowledge of the donor intruding upon their perception of, and feeling of connectedness to, their child. Mothers who used this strategy also tended to create for themselves a picture of an uninterested, indifferent donor, to reduce the level of perceived threat inherent in identity-release:

‘I don’t think the person would want to meet up to be perfectly honest. I can’t imagine that the egg donor would actually want to meet her’. (Lilian)

Amongst mothers who accepted the possibility of contact between their child and the donor, there was a tendency to frame contact with the donor in purely functional terms and in doing so, reject the prospect of having a relationship with the donor. Some mothers also distanced themselves from the identity-release process itself, particularly by situating the identity-release process in the future. Several mothers said that the identity-release process was still too distant a prospect to concern themselves with, embracing an ‘out of sight, out of mind’ attitude. Others distanced themselves from the idea of their child wanting to initiate donor–child contact. This ranged from expressing a hope that the child would not wish to initiate contact, to the overt desire to be absent from the process:

‘It’s up to him really whether he wants to find out really, hopefully I’ll be dead and buried by then [laughs], by the time he’s interested’. (Georgina)

#### Strategy subtheme: reluctant engagement

Some mothers who perceived identity-release as a threat approached it with a certain level of resignation; typically, mothers asserted the right of their child to request the identity of their donor, despite their own negative feelings about it and subsequently reluctantly engaged with the prospect of identity-release. Mothers’ feelings ranged in strength, from feeling that ‘*I wouldn’t particularly relish it, but I would also completely understand it*’, to expecting to feel ‘*utterly broken-hearted*’ by their child’s interest in the donor. Some mothers expressed a resignation to disclosing the details of identity-release to their children, despite a strong preference not to. These attitudes were based on both mothers’ sense of obligation to their child, and to beliefs that, as information about identity-release is ‘*so accessible*’ in a digital information age, the ‘*sensible thing*’ would be to inform their child of identity-release, despite their own, sometimes quite marked, concerns:

‘At some point, we’ll have to have that conversation with her and with [sibling]. But again, I don’t really want to have that. I know she’s entitled to it, and it’s splashed all over her notes so she’s going to find out, but if there was any way of her not finding out I would do that. I would do anything for her not to find that out.’ (Martha)

Several mothers suggested that, should their child ever contact the donor, they would wish to be involved in the process despite their negative feelings about identity-release. Some mothers explained this as a need to be protective of the child, whereas others characterized their involvement as protective of the mother–child relationship:

‘I think if [child] was in touch with [the donor] then I would want to at least have some sort of distal contact about that like I wouldn’t-, I would feel uncomfortable if [child] got in touch with her and then just didn’t tell me anything and that that was a secret.’ (Delphine)

### Acceptance: it is what it is

The second theme describes a small subgroup of mothers’ narratives that displayed few feelings of perceived threat but stopped short of embracing the prospect of future donor–child contact. Mothers demonstrated an acceptance of identity-release, which ranged from expressions of indifference, through to becoming accepting of the realities of identity-release over time. Responding to questions about how they felt about future donor–child contact, these mothers responded with neutral responses such as ‘*that’s fine*’ and ‘*I don’t mind*’. Some mothers referred to their knowledge of identity-release, and how this helped them accept it. Similarly, some recounted scenarios wherein they had intentionally sought out more information about identity-release from healthcare professionals, which in turn led to a greater acceptance of identity-release. Several mothers, like Marie, expressed the feeling that they had come to terms with identity-release over time:

‘Well I don’t feel like threatened by it, I would be quite happy if she wanted to so … if it comes to it and she wants to find out more then yeah. I don’t feel, I wouldn’t say ‘oh no, no, you can’t do that.’ If she wants to then she will … I’m probably a bit more chilled out about it now than I was’.

#### Strategy subtheme: child-led approach

A strategy related to mothers’ acceptance of identity-release was to take a child-led approach to donor–child contact. When asked about their child initiating the identity-release process, mothers tended to respond that it was up to their child. In doing so, mothers deprioritized their own feelings about the prospect of donor–child contact, and instead assumed a neutral stance whereby they asserted the rights of their child. For example, when asked how they felt about possible donor–child contact, several mothers answered with responses such as, ‘*it’s not my decision really is it, it’s [child]’s decision … it’ll be fine, it’s up to him really*’ (Georgina). Often, part of this strategy was to emphasize the importance of providing support to their child, whatever they chose to do. For example, Bonnie explained that:

‘I’m just going to take it from [child]’s lead and respect whatever he wants to do, because it’s, you know … it’s his call, it’s his call and I just … all I need to do is support him to do whatever he wants to do’.

### Embracing identity-release

Another prominent perspective was that identity-release constitutes an opportunity to be embraced, with mothers expressing positive feelings about potential donor–child contact. This was captured by three subthemes: representing *knowledge of genetic origins as essential to the child*, expressing a *personal desire for information about the donor*, and viewing *identity-release as a gateway to a broader family network.* This embracing attitude was associated with one strategy subtheme whereby mothers framed themselves as potential *facilitators of donor–child contact*.

#### Genetic origins as essential to child

The first subtheme describes a tendency to represent identity-release as a benefit by conferring significance onto their child’s genetic origins. Many mothers emphasized their children’s right to access their donor’s identifying information, stating that ‘*it would be wrong to keep it from*’ their children and that their children had a right to know ‘*where they came from*’. Some mothers viewed the donor’s identity as information that fundamentally belonged to their child:

‘Although they’ll have me as their mum, genetically there’ll always be, you know, some things, questions they might ask or just information that they want about themselves, and I think it’s important that, you know, they have access to that information’. (Gabby)

Others stressed that knowledge of their genetic origins was essential for proper identity development and suggested that, without it, their child would ‘*feel that there’s something missing*’ As such, identity-release represented an opportunity for their child to seek information ‘*for their own identity*’. Some mothers expressed regret that the donor’s information was not available to their child at a younger age, stressing the importance of their child knowing ‘*who they’re part of*’ as ‘*a matter of course*’. Similarly, Henrietta reflected on the importance of her own experiences with her family for her own identity development, drawing comparisons between her own and her children’s situation:

‘This is something they need to know, it’s for their future when they grow. I come from quite a mixed family … and part of, for me, growing up was getting to know my half family. So I kind of think of it in those terms, that actually it probably is going to be, for them, making sense of themselves and where they came from’.

#### A personal desire for contact

The majority of mothers who viewed identity-release as an opportunity expressed a desire to have a level of contact with the donor. Mothers reported being ‘*intrigued*’ about the donor’s identity and several mothers expressed a desire to meet the donor. For example, Jemima explained that she wanted ‘*probably more than just to know who she is, but to actually meet her*’; these mothers expressed clear enthusiasm towards the possibility of meeting the donor, describing it variously as a ‘*nice*’ and ‘*exciting*’ prospect. The desire to meet the donor was sometimes intense; one mother recounted her experience of trying to resist the temptation of attempting to trace the donor using online databases and social media platforms, before ultimately accepting that it was up to her children to decide. This was echoed by other mothers who, whilst acknowledging that contacting the donor was their child’s decision, admitted that they’d be ‘*disappointed if [child] doesn’t [contact the donor], because I can’t make that decision, but I would like to meet the person*’*. (Hannah).*

Some mothers also gave distinct reasons why they were interested in contact with the donor. In some cases, they wished to get to know the donor to discover if there were similarities between their child and the donor or donor siblings. Other mothers expressed a wish to meet the donor so that they could ask about their medical history. Most mothers who expressed a wish to contact the donor were motivated by a desire to express gratitude to the donor. This ranged from somewhat cursory references to thanking the donor, to a heartfelt desire to acknowledge the ‘*magnitude*’ of the donor’s contribution. For example, Susanne commented that ‘*I probably would [want to meet the donor] because it would be, you know, I’d want them to see [child] really to see what, you know, what they’ve given us’*.

#### Identity-release as the gateway to a broader family network

An important feature of identity-release donation for many mothers who expressed an ‘embracing’ attitude was the potential for accessing a wider family network. For some mothers, the value of identity-release was not only in enabling their child to contact the donor but also to trace same-donor offspring. Some mothers spoke of their own interest in finding out about same-donor offspring, representing it variously as a ‘*slightly fascinating*’ and ‘*exciting*’ prospect. Other mothers focussed on the importance of tracing same-donor offspring for their child’s benefit, discussing the opportunity to do so as ‘*a very important thing*’ for their child.

A minority of mothers viewed the donor and donor siblings as a potential support network in the event of their own death, often after reflecting on their own older age as parents. One mother observed that it would be ‘*tragic*’ if she and her husband were to die without their child having made contact with the donor, as she anticipated that ‘*[child] would need contact then … and there might be somebody there who’s got a little space for her*’. Another mother went as far as to say that the donor could represent ‘*some kind of parent figure*’ to her child in the case of her death.

#### Strategy subtheme: mother as facilitator

Embracing the prospect of identity-release was associated with one distinct strategy for managing these positive feelings in day-to-day life. Mothers viewed themselves as uniquely placed to facilitate donor–child contact and saw it as a responsibility of egg donation parenthood to help their child have as positive an experience as possible. For some mothers, the very choice to undergo treatment in the UK was a conscious and active one that was based on the perceived benefits of the identity-release system. These mothers often compared UK treatment favourably to the policies governing egg donation in other countries where donors are anonymous. Giving their child the opportunity to trace the donor was seen as taking proper care of their child:

‘I always think about, you know, a lot of women went to [country] and were getting egg donations in [country] and were going there specifically because [country’s] law keeps the details of donors anonymous and then I’m just thinking you couldn’t do that to a child, you know, that’s part of them, but they’re never ever to know or never ever to find out must be really difficult you know? So I think, you know, I think it’s important that they do know that it’s you know, just looking after them as well’. (Gabby)

Mothers also expressed their desire to facilitate the identity-release process by pursuing additional information about the donor. One mother, when explaining why she chased her clinic for a letter the donor had written, observed that she was motivated by the desire to have information in case her child asked for it in the future. This desire to provide their child with plentiful information was shared by several mothers, and doing so was often considered to be an important part of their role as parents:

‘Just whatever details he wants, I’ll provide them … once he’s eighteen, if he wants to contact the donor, then I’ll be able to facilitate that. I’ll guide him in the right direction’. (Roberta)

Additionally, some mothers expressed their intention not only to inform their child of their right to access identifying information about the donor, but to encourage them to do so, due to the belief that it would benefit their child. Moreover, some mothers viewed it as their responsibility to equip their child with the personal and emotional skills necessary to cope with the identity-release process in the future:

‘You hope that you could say, “I’ll give you the skills now to go your own way and make your own decisions”. So I think when the time comes then, you know, my job and [partner]’s job is to lay the groundwork so that when the opportunity arises for some contact … that that can be a wholesome, balanced thing, you know? So [she] can explore her relationships in an unconfusing way’. (Tabitha)

## Discussion

Unexpectedly, around one-third of both mothers and fathers did not understand identity-release donation. Parents were either uncertain about what, if anything, their child could learn about the donor in the future, or they thought that they had used a fully anonymous egg donor to conceive. It is plausible that these parents’ focus had been on falling pregnant after, often, many years of trying, and they did not have the psychological resources available to research the specifics of egg donation legislation. In a Dutch study, many parents of donor-conceived children reported that they had not considered issues of disclosure or future donor–child contact during the treatment process (Visser *et al.*, 2016). Only after childbirth did these topics become pertinent, and many parents wished that they had been able to access practical, professional advice once their child had been born. Currently, the Human Fertilisation and Embryology Authority (HFEA), the UK’s regulatory body, recommends that clinics offer one counselling session to prospective parents prior to treatment with donor gametes (Human Fertilisation and Embryology Authority, 2019). However, follow-up care is not standard practice. A recent review of gamete donation counselling suggests the need for a shift away from a focus on the psychological evaluation of prospective parents, towards a psycho-educational approach that utilizes a combination of information sharing and strategy building throughout the family life course (Crawshaw and Daniels, 2019). It is likely that some of the parents in the present study would have benefitted from an extended period of counselling after childbirth, or from a more diverse selection of formats for accessing information about raising a donor–conceived child, such as workshops on disclosure and donor–child contact (Indekeu and Lampic, 2021).

This study is the first to explore UK egg donation mothers’ perspectives on identity-release donation when children are in early childhood, and the findings offer insights into the complex nature of navigating non-genetic parenthood, as well as the specific challenges of parenting after using identity-release egg donation. The findings suggest that mothers use multiple strategies to make sense of, and manage their feelings about, their use of identity-release egg donation in day-to-day life. These findings are in line with the limited evidence from studies of families formed through identity-release sperm donation, with some parents variously demonstrating comfort with, concerns about, and ambivalent feelings towards future donor–child contact (Isaksson *et al.*, 2016; Widbom *et al.*, 2021).

That a substantial number of mothers perceived identity-release donation as threatening is important and suggests that for some mothers, identity-release contributes to a perception of the donor as an ongoing and salient presence that may put pressure on relationships within the family unit. This finding is consistent with studies of mothers’ motivations for choosing anonymous egg donors, where mothers expressed a preference for anonymous donation to protect the mother–child relationship (Laruelle *et al.*, 2011; Rubin *et al.*, 2015). The coping strategy whereby some mothers distanced themselves from the donor has also been observed in studies investigating mothers’ motivations for choosing anonymous egg donors, in that anonymous donation was chosen to establish and maintain explicit boundaries between the donor and the recipient family, and to limit the donor’s perceived intrusion into family life (Hershberger *et al.*, 2007; Stuart-Smith *et al.*, 2012; Hudson, 2020). The minimization of the donor’s contribution has also been found to aid the parental claiming and bonding process for mothers of identity-release egg donation infants (Imrie *et al.*, 2020). The present study demonstrates that identity-release donation is still felt as a destabilizing force by some mothers well into childhood.

Egg donation mothers’ narratives demonstrated considerable ambivalence about the prospect of future donor–child contact, with mothers expressing complex and sometimes contradictory feelings. Many mothers expressed concern that the future relationship between their child and the donor may be stronger or more meaningful than their own relationship with their child. In the first phase of the present study, some mothers expressed concern that the donor would want to claim the child in the future (Imrie *et al.*, 2020). Some of these mothers also reported that it took time to, or that they were yet to, fully feel that they were the infant’s ‘real’ mother. Parents of children born through identity-release sperm donation have similarly expressed concern about whether donor–child contact would make their children question the authenticity of the father–child relationship (Isaksson *et al.*, 2016). These concerns appear to reflect a belief that genetic relatedness confers upon the biological parent a natural affinity with the child that is ‘given’, and that trumps a non-genetic connection that must be ‘made’ over time (Carsten, 2004). These perspectives are reflective of the sociological concept of ‘genetic thinking’, whereby the broad cultural frame of biogenetic relatedness in the family plays out in how people approach family life (Edwards and Strathern, 2000; Nordqvist, 2017). The effects of such genetic thinking may be compounded by the geneticization of the family in recent years due to advances in biomedical technology (Clarke *et al.*, 2010; Freeman, 2015). Factors such as these may pose a particular challenge to egg donation mothers due to conceptualizations of female reproduction as a natural unity between conception, pregnancy and birth, with the egg as ‘inalienable’ from the mother (Melhuus, 2012). It is therefore possible that mothers of children born through identity-release egg donation, in particular, may have to engage with, and potentially struggle with, the discourse of genetic thinking, and navigate through it to secure their role as a parent (Nordqvist, 2017).

Mothers’ emphasis of the importance of the parenting experience has been acknowledged elsewhere in the identity-release donation literature. Imrie and colleagues (2020) found that egg donation mothers minimized the role of the donor, whilst emphasizing their own characteristics as an important factor in their infant’s developing personality. Similarly, Widbom *et al.* (2021) found that fathers of adult children conceived via sperm donation vacillated between emphasizing the importance of ‘doing’ parenthood and the difficulty of not ‘being’ the genetic father. This tension between ‘doing’ parenthood and legitimately ‘being’ a mother was evident in the present study: some mothers expressed concerns about their child accessing the donor’s identifying information, but they reassured themselves with the possibility that developing a strong mother–child bond over time would mean their child would be less interested in obtaining the donor’s identity in the future.

Mothers who viewed identity-release donation as an opportunity to be embraced often positioned themselves as facilitators of donor–child contact. A similar role has been reported amongst mothers of children born through identity-release sperm donation; when discussing their approach to the disclosure process, mothers represented themselves as ‘process managers’, demonstrating the belief that a responsibility of gamete donation parenthood is to initiate the disclosure process and keep it moving forward (Isaksson *et al.* 2016). Parallels can be drawn between this attitude and adoption communication openness, wherein parents explore the meaning of adoption within their lives, facilitate discussion about adoption in an emotionally supportive environment, and potentially facilitate contact between the birth and adoptive families (Brodzinsky, 2005). This subset of mothers in the present study seemed particularly comfortable with, and oriented towards, acknowledging their child’s dual connection to both their own family and the donor. Whether this attitude is associated with differences in family functioning warrants future empirical verification.

Mothers who felt positively about their child discovering the donor’s identity considered the donor’s identity as essential information for their child to develop a full and positive self-concept. This is in line with theories of identity development that position knowledge of one’s biological origins as an essential factor that contributes to identity development (Erikson, 1980). Donor-conceived people who are denied this information are deprived of one key component of self-knowledge (Benward, 2012). This perspective has been observed amongst identity-release sperm donation parents, with some mothers ascribing a biopsychosocial role to the donor by designating shared physical or personal characteristics as identity-relevant information (Goldberg and Scheib, 2015; Widbom *et al.*, 2021). The present study demonstrates that some egg donation mothers, too, conceptualize the donor’s identity as identity-relevant information for their children.

A further noteworthy finding is the representation of identity-release by some mothers as a gateway to a broader family network. Most commonly, mothers represented identity-release donation as a link to a network of same-donor offspring. This was often, but not always, discussed in the context of their child being an only child, and accompanied by the hope that a network of same-donor offspring might provide support and comfort to their child in the future. Research from the broader parenting literature also attests to concerns amongst many parents about raising only-children, with siblings often positioned as providing children with the social skills and support necessary for optimal adjustment (Veenhoven and Verkuyten, 1989; Coan *et al.*, 2018). Within the donor conception literature, recipient parents and donors in some family types have similarly been found to characterize embryo and sperm donation in terms of developing a network of extended family bonds (Goedeke *et al.*, 2015; Frith *et al.*, 2017; Widbom *et al.*, 2021). The present study adds to this literature by showing that some egg donation mothers conceptualize identity-release donation as potentially providing their child with an extended family network.

The present sample consisted primarily of *cis*-gender, heterosexual, White, highly educated couples; the findings will therefore have limited generalizability to populations of different gender identities, sexual orientations, ethnicities or socioeconomic statuses. For example, LGBTQIA+ identifying parents have been found to form, make meaning of and enact relationships with gamete providers in qualitatively different ways to *cis*-gender heterosexual identifying parents (Nordqvist, 2014; Provoost *et al.*, 2018). However, as egg donation treatment is costly, and funding for treatment is limited (Human Fertilisation and Embryology Authority, 2020), IVF and egg donation are generally less accessible to patients of a lower socioeconomic status. Thus, although homogenous, the current sample is representative of the *cis*-gender, heterosexual couples who can currently access ARTs in the UK (Rubin *et al.*, 2015). It was also the case that, of the families who were uncontactable or declined to participate again at phase two of the present study, 77% had planned not to, or were unsure whether to, tell their child about their method of conception. Consequently, the present sample may over-represent participants who favoured disclosure and who felt positive about donor–child contact. Previous studies have found that parents of children born through sperm donation who declined to take part in research tended to do so to keep the nature of the child’s conception a secret, and to protect the parents from reminders of their infertility (Cook *et al.*, 1995). This may also be the case with the non-participants in the current study.

Current HFEA guidelines state that clinics should inform parents of the fact that their child will be able receive identifying information about the donor, and they recommend that this be shared with the child at an early age. However, the findings of this study suggest that there is still a considerable lack of awareness regarding accessing information about the donor, and about the possibility of donor–child contact in the future. A significant proportion of mothers viewed the donor as threatening 5 years after the birth of their child. This suggests that, for some mothers, the challenges of parenting a child conceived using an identifiable egg donor may not be limited to the pre- and post-natal periods, and that feelings of threat, opportunity and ambivalence may develop and subside over the family life course. Rather than simply encouraging parents to access additional information about the donor, clinics may better serve their patients by also acknowledging and normalizing the possibility that parents may feel ambivalence towards the availability of donor information and the prospect of donor–child contact. Patients seeking treatment with donor gametes in the UK may benefit from the provision of counselling following the birth of their child.

## Data Availability

The data underlying this article cannot be shared publicly in order to protect the privacy of the individuals that participated in the study.
